# Influence of geographic origin on AIDS and serious non-AIDS morbidity/mortality during cART among heterosexual HIV-infected men and women in France

**DOI:** 10.1371/journal.pone.0205385

**Published:** 2018-10-31

**Authors:** Laure-Amélie de Monteynard, Sophie Matheron, Sophie Grabar, Pierre de Truchis, Jacques Gilquin, Juliette Pavie, Odile Launay, Jean-Luc Meynard, Marie-Aude Khuong-Josses, David Rey, Aba Mahamat, Rosemarie Dray-Spira, Anne Simon, Dominique Costagliola, Sophie Abgrall, on behalf of the FHDH-ANRS CO4

**Affiliations:** 1 Sorbonne Universités, UPMC Université Paris 06, INSERM, Institut Pierre Louis d’épidémiologie et de Santé Publique (IPLESP UMRS 1136), Paris, France; 2 Sorbonne Paris cité, Université Paris Diderot, UMR 1137, Paris, France; 3 AP-HP, Hôpital Bichat-Claude Bernard, Service des Maladies Infectieuses et Tropicales, Paris, France; 4 Université Paris Descartes, Sorbonne Paris cité, Paris, France; 5 APHP, Groupe Hospitalier Cochin Broca Hôtel-Dieu, Unité de Biostatistique et Epidémiologie, Paris, France; 6 AP-HP, Hôpitaux Universitaires Paris-Ile de France-Ouest, Hôpital Raymond-Poincaré, Département de Médecine Aigüe Spécialisée, Garches, France; 7 AP-HP, Hôpital Hôtel-Dieu, Unité d’immunoinfectiologie, Paris, France; 8 AP-HP, Hôpital Européen Georges Pompidou, Service d’immunologie clinique, Paris, France; 9 Université Paris Descartes, Sorbonne Paris cité, Paris, France; 10 AP-HP, Hôpital Cochin, Paris, France; 11 AP-HP, Hôpital Saint Antoine, Service des Maladies Infectieuses et Tropicales, Paris, France; 12 Centre Hospitalier Saint-Denis, Hôpital Delafontaine, Service des Maladies Infectieuses et Tropicales, Saint-Denis, France; 13 Hôpitaux Universitaire Strasbourg, Centre de Soins de l’Infection par le VIH, Strasbourg, France; 14 Centre Hospitalier Andrée Rosemon, Service des Maladies Infectieuses et Tropicales, Cayenne, France; 15 AP-HP, Groupe Hospitalier Pitié-Salpêtrière, Département de médecine interne, Paris, France; 16 AP-HP, Hôpital Antoine Béclère, Service de Médecine interne/Immunologie clinique, Clamart, France; Katholieke Universiteit Leuven Rega Institute for Medical Research, BELGIUM

## Abstract

**Background:**

The influence of geographic origin on the risk of severe illness and death on cART has not been explored in European countries.

**Method:**

We studied antiretroviral-naïve heterosexual HIV-1-infected individuals enrolled in the FHDH-ANRS CO4 cohort in France who started cART between 2006 and 2011. Individuals originating from France (French natives), sub-Saharan Africa (SSA) and non-French West-Indies (NFW) were studied until 2012. Crude and adjusted rate ratios (aRR) of severe morbid events/deaths (AIDS-related and non-AIDS-related) were calculated using Poisson regression models stratified by sex, comparing each group of migrants to French natives.

**Results:**

Among 2334 eligible men, 1379 (59.1%) originated from France, 838 (35.9%) from SSA and 117 (5.0%) from NFW. SSA male migrants had a higher aRR for non-AIDS infections, particularly bacterial infections (aRR 1.56 (95% CI 1.07–2.29), p = 0.0477), than French natives. Among 2596 eligible women, 1347 (51.9%) originated from France, 1131 (43.6%) from SSA, and 118 (4.5%) from NFW. SSA and NFW female migrants had a higher aRR for non-AIDS infections, particularly non-bacterial infections (respectively, 2.04 (1.18–3.53) and 7.87 (2.54–24.4), p = 0.0010), than French natives. We observed no other significant differences related to geographic origin as concerns the aRRs for AIDS-related infections or malignancies, or for other non-AIDS events/deaths such as cardiovascular disease, neurological/psychiatric disorders, non-AIDS malignancies and iatrogenic disorders, in either gender.

**Conclusion:**

Heterosexual migrants from SSA or NFW living in France have a higher risk of non-AIDS-defining infections than their French native counterparts. Special efforts are needed to prevent infectious diseases among HIV-infected migrants.

## Introduction

In high-income countries, HIV-infected adults with controlled infection and high CD4 cell counts currently live longer than ever before [1], and the main causes of morbidity and mortality have switched from AIDS-defining to non-AIDS-defining disorders [2,3,4,5,6,7,8,9]. In western European countries, despite experiencing more AIDS-defining events [10,11], migrants from sub-Saharan Africa (SSA) have lower mortality than non-migrants, mainly owing to lower non-AIDS mortality [12]. In France, heterosexual migrant men originating from SSA and non-French West Indies (NFW), and non-migrant men, have a higher risk of new AIDS-defining events, serious non-AIDS events or death after cART initiation than non-migrant men who have sex with men (MSM), while this is not the case of women [9]. This latter study compared heterosexual individuals to MSM, two groups with different socioeconomic and lifestyle factors, and specific causes of morbidity were not assessed. In addition, no previous study has examined the specific morbidity of migrants compared to non-migrants. We therefore described and compared causes of severe morbidity and mortality between SSA or NFW migrant and non-migrant heterosexual HIV-infected men and women between 2006 and 2012.

## Methods

### Individuals

Created in 1989, the French Hospital Database on HIV (FHDH, ANRS CO4) is a large prospective cohort of HIV-infected individuals receiving care in one of 70 French participating hospitals, collecting standardized clinical, therapeutic and biological variables at each outpatient visit or hospital admission and/or at least every 6 months [13]. The only enrolment criteria are documented HIV-1 or HIV-2 infection, follow-up in a FHDH participating centre, and written informed consent. Data submitted by the participating centres to the data coordinating and analysis centre are anonymized, then encrypted. The FHDH was approved by the French data protection agency (Commission Nationale de l'Informatique et des Libertés) on 27 November 1991 (Journal Officiel, 17 January 1992). For the present study, we selected antiretroviral-naive heterosexual HIV-1-infected individuals from the FHDH, aged at least 16 years, who started a first combined antiretroviral therapy (cART) between 1 January 2006 and 31 December 2011, at least one year prior to the database update (31 December 2012). Individuals were excluded if their first cART regimen was prescribed for pregnancy. Patients had to have at least one CD4 cell count and one plasma viral load (pVL) measurements within 6 months prior to treatment initiation and one CD4 cell count and one pVL measurements following cART initiation. Migrant status was based on the United Nations definition, as follows: “anyone born and having lived outside France and now residing in France, whatever their nationality and the duration of stay in France” [14]. All individuals originating from France, including the French West Indies (Martinique, Guadeloupe, French Guyana), SSA or NFW, were considered.

### Coding causes of severe events

Severe events included any AIDS-defining event or death from AIDS [15] and any serious non-AIDS event or death from causes other than AIDS occurring between cART initiation and 31 December 2012. A serious non-AIDS event was defined as the primary underlying cause of any unscheduled hospitalization for more than 24 hours when an AIDS event was not the reason for hospitalization. If more than one non-AIDS event was notified for one hospitalization with no main reason notified in the discharge file, the most severe event was chosen as the cause for hospitalization in a hierarchical way: non-AIDS cancer, cardiovascular event, end-stage renal failure, acute liver failure, suicide attempt, infection, iatrogenic systemic event, then other systemic event. Hospitalization for a non-morbid cause (routine check-up, chemotherapy, blood transfusion, etc.) was not considered. If any of the following severe morbid events for which hospitalization is essential was reported outside the context of an hospitalization, ie non-AIDS-defining cancer, cardiovascular event (stroke, myocardial infarction or coronary revascularization), deep-vein thrombosis or pulmonary embolism, end-stage renal disease (dialysis or renal transplantation), decompensated liver disease, complicated diabetes mellitus, severe bacterial, fungal, viral or parasitic infection, acute neuropsychiatric diseases (e.g. suicide attempt), acute surgical conditions (e.g. gastrointestinal bleeding), it also counted as a serious non-AIDS event as proposed elsewhere [8,16]. All successive events were considered for each patient.

Severe events were categorized according to the Coding of Death in HIV (CoDe) (available at: http://www.cphiv.dk/Portals/0/files/Code%20Protocol%202.3.pdf), adapted to take AIDS and severe non-AIDS events into account, in addition to death. We grouped causes as follows: AIDS-defining infection (CoDe 01.1), AIDS-defining malignancy (CoDe 01.2), non-AIDS bacterial infection (CoDe 02.1), other non-AIDS infection (CoDe 02.2), liver-related disorder (CoDe 03 and 14), non-AIDS malignancy (CoDe 04), cardiovascular disease (CoDe 08, 09 and 24), respiratory disease (CoDe 13 and 25), renal failure and urogenital disease (CoDe 15 and 28), central neurological and psychiatric disorders (CoDe 19, 22 and 23), gastrointestinal disorders (CoDe 10 and 26), skin, rheumatic and peripheral neurological disorders (CoDe 27), haematological disease (CoDe 20), accidental/violent events (CoDe 16 and 17) and endocrine and metabolic disease (CoDe 05, 06 and 21), as proposed elsewhere [2]. In addition, owing to the large number of events, a category of “iatrogenic disorders” was created including any reported event, mainly cutaneous, hematological, hepatic or intestinal, associated with drug toxicity. Other causes not mentioned in this classification, and unknown causes, were classified as “other”. Only categories with more than 20 events/deaths are presented here.

### Statistical analysis

The Chi2 and Kruskal-Wallis tests were used to compare the demographic, immunovirological and clinical characteristics of the individuals at cART initiation, across the three regions of origin, in men and in women. The number of severe events, person-years at risk, and incidence rates (IRs) of severe events (per 1000 person-years of follow-up) were calculated. Person-years of follow-up were calculated from the date of cART initiation to the date of death for known deceased individuals, last follow-up visit for individuals with no follow-up visit during the 6-month period before 31 December 2012 (last database update) or 31 December 2012 for the remainder of the cohort. Crude and adjusted IRs were estimated according to geographic origin, in men and in women. Poisson regression models were used to identify differences in morbidity/mortality according to geographic origin, in men and in women. Incidence rates (IRs) and rate ratios (RRs) were adjusted for the following characteristics at cART initiation: age, region of care (Paris area, other regions of metropolitan France plus the Reunion Island and French West Indies), CD4 cell count, pVL, AIDS status, hepatitis B and/or C infection and smoking status for each category of events/deaths, plus the body mass index for cardiovascular diseases. All analyses were done with SAS v9.4 software (SAS Institute, Inc, Cary, NC). A p value <0.05 was considered to denote statistical significance.

## Results

### Population

A total of 2334 men and 2596 women fulfilled the inclusion criteria for this study. The men’s geographic origin was France in 1379 cases (59.1%), SSA in 838 (35.9%) and NFW in 117 (5.0%). The women’s geographic origin was France in 1347 cases (51.9%), SSA in 1131 (43.6%) and NFW in 118 (4.5%) (Table 1). In each sex, SSA migrants were the youngest individuals and NFW migrants were the oldest. At cART initiation, in men and in women, NFW migrants had the lowest CD4 cell counts and the highest rate of AIDS, while individuals originating from France had the highest CD4 cell counts and the lowest rate of AIDS. Smoking was less prevalent among migrants than among French natives, in both genders.

**Table 1:** Characteristics of the population at cART initiation according to geographic origin in heterosexual men and women (France, 2006–2011, n = 4930).

	Heterosexual men (n = 2334)				Heterosexual women (n = 2596)			
	France^a^	SSA	NFW		France^a^	SSA	NFW	
	N = 1379	N = 838	N = 117	P^b^	N = 1347	N = 1131	N = 118	P^b^
**Age [years, median (IQR)]**	43.7 (36.2–51.6)	40.3 (34.5–47.1)	45.4 (37.9–52.0)	<0.0001	38.6 (31.1–47.7)	34.3 (29.4–40.3)	42.1 (34.4–49.1)	<0.0001
**Period of cART initiation [n (%)]**								
2006–2007	553 (40.1)	338 (40.3)	77 (65.8)		511 (37.9)	465 (41.1)	73 (61.9)	
2008–2011	826 (59.9)	500 (59.7)	40 (34.2)	<0.0001	836 (62.1)	666 (58.9)	45 (38.1)	<0.0001
**Region of care [n (%)]**								
Paris area	584 (42.4)	650 (77.6)	45 (38.4)		660 (49.0)	826 (73.0)	45 (38.1)	
Southeast France	162 (11.7)	27 (3.2)	0 (0)		166 (12.3)	32 (2.8)	0 (0)	
Other/Reunion island	509 (36.9)	161 (19.2)	3 (2.6)		448 (33.3)	269 (23.8)	1 (0.9)	
French West Indies	124 (9.0)	0 (0)	69 (59.0)	<0.0001	73 (5.4)	4 (0.4)	72 (61.0)	<0.0001
**CD4 cell count [n (%)]**								
<200	523 (37.9)	348 (41.5)	55 (47.0)		412 (30.6)	399 (35.3)	47 (39.8)	
≥200–349	527 (38.2)	331 (39.5)	43 (36.7)		578 (42.9)	488 (43.2)	53 (44.9)	
≥350–499	222 (16.1)	121 (14.4)	11 (9.4)		251 (18.6)	178 (15.7)	12 (10.2)	
≥500	107 (7.8)	38 (4.5)	8 (6.8)	0.0159	106 (7.9)	66 (5.8)	6 (5.1)	0.0108
**CD4 cell count [median (IQR)]**	250 (122–342)	231 (120–321)	214 (56–299)	0.0008	270 (171–357)	251 (154–333)	235 (148–313)	0.0004
**pVL [log10 copies/mL, median (IQR)]**	4.88 (4.30–5.35)	4.84 (4.26–5.34)	4.92 (4.30–5.39)	0.7363	4.57 (3.88–5.10)	4.58 (3.91–5.11)	4.64 (3.99–5.10)	0.8677
**AIDS status [n (%)]**	230 (16.7)	164 (19.6)	34 (33.3)	<0.0001	166 (12.3)	171 (15.1)	22 (18.6)	0.0400
**Hepatitis B antigen [n (%)]**	53 (3.8)	89 (10.6)	4 (3.4)	<0.0001	34 (2.5)	75 (6.6)	4 (3.4)	<0.0001
**Hepatitis C antibodies [n (%)]**	36 (2.6)	20 (2.4)	3 (2.6)	0.9480	48 (3.6)	38 (3.4)	0 (0)	0.1156
**Smoking status [n (%)]**								
Never	281 (20.4)	375 (44.7)	63 (53.8)		337 (25.0)	701 (62.0)	61 (51.7)	
Previous	151 (10.9)	75 (9.0)	7 (6.0)		56 (4.2)	27 (2.4)	3 (2.5)	
Current	411 (29.8)	175 (20.9)	14 (12.0)		221 (16.4)	69 (6.1)	8 (6.8)	
Unknown	536 (38.9)	213 (25.4)	33 (28.2)	<0.0001	733 (54.4)	334 (29.5)	46 (39.0)	<0.0001
**Body mass index [n (%)**								
<18	11 (0.8)	15 (1.8)	0 (0)		49 (6.6)	19 (1.7)	3 (2.5)	
≥18–30	1024 (74.3)	653 (77.9)	59 (50.4)		911 (67.6)	835 (73.8)	51 (43.2)	
>30	69 (5.0)	70 (8.4)	4 (3.4)		140 (10.4)	120 (10.6)	4 (3.4)	
Unknown	275 (19.9)	100 (11.9)	54 (46.2)	<0.0001	247 (18.3)	157 (13.9)	60 (50.9)	<0.0001
**Follow-up time after cART initiation**								
**[years, median (IQR)]**	2.6 (1.4–4.0)	2.6 (1.4–4.1)	2.3 (1.9–3.6)	0.1636	2.6 (1.5–4.0)	2.7 (1.6–4.1)	1.8 (1.0–3.5)	<0.0001

Data are counts (proportions) and median (interquartile range)

^**a**^Including French West Indies (Martinique, Guadeloupe, French Guyana)

^**b**^The Chi2 and Kruskal-Wallis tests were used to compare the demographic, immunovirological and clinical characteristics of the individuals

Abbreviations: SSA, Sub-Saharan Africa; NFW, Non-French West-Indies; cART, combination antiretroviral

### Incidences and risks of severe events in men

During 6524 person-years of follow-up, a total of 771 severe events, including 27 deaths, were recorded in 495 men, of whom 299 were French natives, 167 SSA migrants and 29 NFW migrants. Overall, crude IRs of severe events were 118.7 per 1000 person-years for French natives, 116.4 for SSA migrants and 125.8 for NFW migrants. Adjusted IR of severe events was highest among SSA migrants (183.6 in French natives, 226.4 in SSA migrants and 163.1 in NFW migrants), with an adjusted rate ratio (aRR) of severe events among SSA migrants estimated as 1.23 (95% confidence interval (CI): 1.04–1.47) compared to French natives. When AIDS events and severe non-AIDS events were considered separately, SSA migrants had a higher aRR of both AIDS events (1.38 (95%CI: 0.96–1.99)) and non-AIDS events (1.19 (95%CI: 0.98–1.44)) than French natives. Within the different non-AIDS events categories, there was a higher aRR of non-AIDS infections among SSA migrants (1.58 (95%CI: 1.15–2.15)) than among French natives, due to a higher aRR of non-AIDS bacterial infections in SSA migrants than in French natives (Table 2). The main non-AIDS bacterial infection was acute pneumonia, with 61 cases (40.9%). No other significant differences were observed in the aRRs of cardiovascular diseases, neurological/psychiatric disorders, non-AIDS malignancies or iatrogenic effects according to geographic origin.

**Table 2:** Crude and adjusted incidence rates (IRs) per 1000 person-years (PY) and 95% confidence intervals (95% CI)), and crude and adjusted rate ratios (RRs) and 95% CI of clinical events/deaths by geographic origin, in heterosexual men and women (Poisson regression, n = 4930, France, 2006–2012).

		Heterosexual men (n = 2334)						Heterosexual women (n = 2596)					
Clinical events	Geographic	People	Events	Crude	Adjusted^a^	Crude	Adjusted^a^	People	Events	Crude	Adjusted^a^	Crude	Adjusted^a^
	origin	with		IRs per 1000 PY	IRs per 1000 PY	RRs (95%CI)	RRs (95%CI)	with		IRs per 1000 PY	IRs per 1000 PY	RRs (95%CI)	RRs (95%CI)
		event(s)				p-value	p-value	event(s)				p-value	p-value
**AIDS infections**	France	71	87	22.4	26.3	1	1	43	51	13.6	10.3	1	1
	SSA	49	54	22.9	34.0	1.02 (0.73–1.43)	1.29 (0.86–1.94)	47	55	16.7	11.8	1.23 (0.84–1.80)	1.15 (0.75–1.77)
	NFW	8	11	37.4	23.6	1.67 (0.89–3.12)	0.90 (0.44–1.82)	5	5	18.5	20.4	1.36 (0.54–3.42)	1.97 (0.70–5.60)
						0.3246	0.4168					0.5180	0.4450
**AIDS**	France	19	19	4.9	5.3	1	1	8	8	2.1	3.5	1	1
**malignancies**	SSA	14	14	5.9	9.5	1.21 (0.61–2.42)	1.78 (0.79–4.01)	11	11	3.3	8.0	1.57 (0.63–3.90)	2.31 (0.81–6.61)
	NFW	1	1	3.4	3.0	0.69 (0.09–5.18)	0.56 (0.06–4.98)	0	0	**-**	**-**	**-**	**-**
						0.7724	0.2896					0.3051	0.1291
**Non-AIDS**	France	71	77	19.9	29.1	1	1	55	59	15.7	31.6	1	1
**bacterial**	SSA	53	62	26.3	45.4	1.33 (0.95–1.85)	1.56 (1.07–2.29)	36	41	12.4	34.5	0.79 (0.53–1.18)	1.09 (0.70–1.71)
**infections**	NFW	10	10	34.0	49.5	1.71 (0.89–3.31)	1.70 (0.82–3.56)	6	6	22.2	50.6	1.41 (0.61–3.28)	1.60 (0.58–4.39)
						0.1253	0.0477					0.3102	0.6502
**Other non-AIDS**	France	38	39	10.0	16.4	1	1	29	29	7.7	4.7	1	1
**infections**	SSA	31	31	13.2	26.0	1.31 (0.82–2.10)	1.59 (0.93–2.72)	32	35	10.6	9.0	1.37 (0.84–2.25)	2.04 (1.18–3.53)
	NFW	3	3	10.2	12.6	1.01 (0.31–3.28)	0.77 (0.21–2.83)	6	6	22.2	36.0	2.88 (1.19–6.93)	7.87 (2.54–24.4)
						0.5332	0.1957					0.0790	0.0010
**Cardiovascular**	France	36	42	10.8	7.3	1	1	25	27	7.2	11.9	1	1
**diseases**	SSA	19	20	8.5	7.9	0.78 (0.46–1.33)	1.08 (0.59–1.95)	10	10	3.0	5.8	0.42 (0.20–0.87)	0.49 (0.21–1.13)
	NFW	3	3	10.2	11.4	0.94 (0.29–3.04)	1.56 (0.44–5.55)	0	0	**-**	**-**	**-**	**-**
						0.6608	0.8016					0.0121	0.0240
**Central**	France	27	30	1.8	1.4	1	1	24	25	4.3	7.8	1	1
**neurological and**	SSA	8	8	0.4	0.6	0.24 (0.03–1.91)	0.40 (0.04–3.60)	17	19	1.8	3.6	0.43 (0.17–1.09)	0.45 (0.16–1.31)
**psychiatric**	NFW	2	2	3.4	4.6	1.88 (0.23–15.30)	3.30 (0.23–47.90)	4	4	7.4	8.8	1.74 (0.40–7.56)	1.12 (0.20–6.31)
**disorders**						0.1997	0.4395					0.1006	0.3042
**Non-AIDS**	France	33	36	9.3	10.9	1	1	21	24	6.4	8.7	1	1
**malignancies**	SSA	9	12	5.1	8.8	0.55 (0.29–1.05)	0.80 (0.38–1.67)	9	9	2.7	5.9	0.43 (0.20–0.92)	0.68 (0.29–1.58)
	NFW	1	2	6.8	7.6	0.73 (0.18–3.04)	0.70 (0.15–3.11)	0	0	**-**	**-**	**-**	**-**
						0.1669	0.7589					0.0208	0.1932
**Iatrogenic effects**	France	20	21	5.4	9.6	1	1	24	26	6.9	7.4	1	1
	SSA	9	9	3.8	7.7	0.71 (0.32–1.54)	0.80 (0.32–2.00)	17	17	5.2	6.3	0.74 (0.40–1.37)	0.85 (0.43–1.71)
	NFW	1	1	3.4	3.7	0.63 (0.08–4.66)	0.38 (0.05–3.28)	1	1	3.7	5.8	0.53 (0.07–3.94)	0.78 (0.09–6.83)
						0.6274	0.5875					0.5504	0.8923

Abbreviations: SSA, Sub-Saharan Africa; NFW, Non-French West-Indies; cART, combination antiretroviral therapy

^**a**^Adjusted for variables at cART initiation: age, the region of care, the CD4 cell count, the pVL, pre-existing AIDS status, hepatitis B virus antigen and/or anti-hepatitis C virus antibody status, and the smoking status for all analyses, plus the body mass index for cardiovascular diseases

### Incidences and risks of severe events in women

During a follow-up of 7324 person-years, a total of 624 severe events, including 48 deaths, were recorded in 413 women, of whom 213 were French natives, 181 SSA migrants and 18 NFW migrants. Overall, crude IRs of severe events were 87.0 per 1000 person-years among French natives, 82.2 among SSA migrants and 96.3 among NFW migrants. Within the different non-AIDS events categories, there was a higher aRR of non-AIDS infections among SSA and NFW migrants (respectively 1.40 (95%CI: 0.99–1.97) and 2.91 (95%CI: 1.36–6.22)) than among French natives, due to a higher aRR of non-AIDS non-bacterial infections (Table 2). The main non-AIDS bacterial infection was acute pneumonia, with 29 cases (27.4%), and the main non-AIDS non-bacterial infections were non-bacterial diarrhea (11 cases, 15,7%), viral meningo-encephalitis (10 cases, 14.3%), herpes zoster (9 cases, 12,8%) and malaria (6 cases, 8.6%). No other significant differences were observed in the aRR of cardiovascular diseases, neurological/psychiatric disorders, non-AIDS malignancies, or iatrogenic disorders according to geographic origin, although SSA migrants had lower crude RRs for cardiovascular and for non-AIDS malignancies events/deaths than French native women.

## Discussion

We found that the likelihood of non-AIDS infections, and particularly bacterial infections, was significantly higher among male migrant from sub-Saharan Africa than among male French natives. The likelihood of non-AIDS infections, particularly non-bacterial infections, was significantly higher among female migrants from sub-Saharan Africa and non-French West Indies than among female French natives.

A recent collaborative cohort study comparing mortality in Western Europe according to geographic origin, sex and HIV transmission category [17] showed that, compared to native heterosexual men, mortality was lower among male heterosexual SSA migrants and similar among male heterosexual Caribbean migrants, whereas, compared to female heterosexual natives, mortality among female heterosexual SSA migrants was similar and mortality among female heterosexual Caribbean migrants was higher. Differences among host countries were not analyzed, despite differences in recruitment to this study, as well as differences in social determinants [18], including access to HIV screening, health care and antiretroviral therapy [19,20]. In addition, for a given country of origin, social determinants and access to medical services might have been influenced by the migrants’ native language(s) [20,21]. Non-AIDS morbidity has not previously been studied according to geographic origin.

Non-AIDS-defining events, and especially bacterial infections, now represent the leading cause of severe morbidity among HIV-infected patients [22,23,24]. Immunosuppression, even when moderate, is associated with an increased risk of bacterial, viral, fungal and parasitic infections [7,25,26]. We found that the risk of infection among heterosexual migrants from SSA or NFW was higher than among their native French counterparts. We could not explore the reasons for this difference, but one explanation might be exposure to infectious pathogens during visits to the country of origin as migrants travel more to the tropics than non migrants in Western Europe [27]. This implies a need for tailored counseling to ensure that migrants travel with enough and adequate medications, including antiretroviral drugs and antimalarial prophylaxis [28,29,30] and that they also have adequate pneumococcal and influenza vaccination, as recommended [31]. Moreover, in addition to lower CD4 cell counts at cART initiation [32], SSA and NWF migrants tends to have lower CD4 cell recovery during the first two years of treatment [9] and might thus be more vulnerable to non-AIDS infections during this period [33].

This study has several limitations. First, no data on treatment adherence are available in the FHDH database. However, we recently showed that in women and in non homosexual men followed in France, SSA and NFW migrants had no difference in time to viral undetectability than their native French counterparts [9]. Moreover, we used an intent-to-continue-treatment approach, meaning that cART interruption, which might have differed among the groups, was not taken into account in the models. However, it is noteworthy that the median number of visits per year and the median time between visits did not differ according to geographic origin or sex (data not shown). Because FHDH is implemented in infectious diseases and internal medicine departments, non-AIDS non-infectious events leading to hospitalization in other departments may have been under-reported, leading to an underestimation of non-AIDS events. Similarly, because a predefined hierarchy was sometimes used to classify morbidity and mortality, misclassification was possible. However, there is no reason to suspect that under-reporting of some non-AIDS events or misclassification between the different clinical categories would have differed according to geographic origin. The main strength of this study is its large size and lengthy follow-up, which allowed us to assess meaningful clinical outcomes, including serious non-AIDS events in the different subgroups.

In summary, after cART initiation, heterosexual men and women from sub-Saharan Africa and non-French West Indies have a higher risk of non-AIDS infections than their native France counterparts. However, we found no other substantial differences in terms of severe AIDS or non-AIDS events/deaths in either gender. Special attention should thus be paid to the prevention of infectious diseases among migrants, including screening of vaccination status and completion of vaccine schedules [34], pre-travel counseling to prevent gastro-intestinal diseases and to prevent malaria with exposure prophylaxis counseling and chemoprophylaxis prescription, and post-travel close monitoring of infectious tropical diseases which could unbalance their care.
